# Nanometer Titanium Dioxide Mediated High Efficiency Photodegradation of Fluazifop-p-Butyl

**DOI:** 10.3390/ijerph16193600

**Published:** 2019-09-26

**Authors:** Guangling Li, Zhiguang Hou, Ruihong Zhang, Xiling Chen, Zhongbin Lu

**Affiliations:** 1 School of Resources and Environmental Sciences, Jilin Agricultural University, Changchun 130118, China; lgl3298@126.com (G.L.); zhiguanghou@aliyun.com (Z.H.); ivy20170309@126.com (R.Z.); 2 School of Resources and Environmental Sciences, Henan Institute of Science and Technology, Xinxiang 453003, China; xilingchen@aliyun.com

**Keywords:** FPB, nano-TiO_2_, photocatalytic degradation, derivatization, GC-MS

## Abstract

The widespread use of fluazifop-p-butyl (FPB) contributes to its presence in the environment. Considering the ecological risks of FPB residues in the environment, the anatase nanometer titanium dioxide (nano-TiO_2_) mediated photocatalytic degradation of FPB was studied by smearing FPB and nano-TiO_2_ together on a glass plane; illumination, trimethylsilane derivatization of photolysis products, high performance liquid chromatography (HPLC) quantitative analysis and gas chromatograph-mass spectrometer (GC-MS) identification were used. Results showed that the first order dynamic model could describe the photodegradation of FPB by nano-TiO_2_ mediated, and the photodegradation and photosensitization rates were found to be positively correlated with the dose of nano-TiO_2_ at lower dose ranges. It is noticeable that a strong photosensitization effect was exhibited on degradation of FPB, not only under high-pressure mercury lamps, but also simulated sunlight (xenon lamp light). Ultimately, twelve main photolytic products were reasonably speculated, whilst five photolysis pathways were proposed. These results together suggest that nano-TiO_2_ can be used as an effective photosensitizer to accelerate FPB photolysis.

## 1. Introduction

The use of chemical pesticides in modern agriculture is vital to guarantee crop yield and quality and, as such, it will continue to be widely applied. However, the intense use of pesticides will inevitably lead to environmental contamination and ultimately constitute threats to human health. Based on their toxic nature, there is considerable concern regarding their environmental risks.

FPB (Fluazifop-p-butyl) is a highly selective systemic, post-emergence aryloxyphenoxypropionate (AOPP) herbicide that is registered for use in selectively controlling both annual and perennial grassy weeds for many crops, but does little or no harm to non-graminaceous crops [1]. FPB is described in the database PPDB (Pesticide Properties Database) as a straw color liquid with a melting point of −46 °C and low water solubility (s = 0.93 mg/L, 20 °C) and volatile (v.p. = 0.12 mPa, 20 °C). It is resistant to hydrolysis at pH 4 and pH 7, but hydrolyzes rapidly at pH 9. The octanol−water partition coefficient (log *P*) of FPB is 4.5 (pH 7, 20 °C), and its Henry’s law constant is 4.9 × 10^−2^ Pa m^3^/moL (25 °C). According to the literature [2,3,4,5,6], AOPPs can pass readily into fish tissue, and is thus highly toxic to aquatic species, particularly fish, and can induce liver toxicity and injury. Furthermore, FPBs may induce pathological changes in testes [7]. The widespread use of AOPPs contributes to their presence in the environmental matrices [8,9]. Therefore, the accumulation of these herbicides may potentially destroy fish populations or elevate the concentration of undesirable toxicants in natural water systems and jeopardize the health of humans.

In the environment, FPB is degraded primarily through hydrolysis with an estimated half-life of one to two weeks. However, it is relatively stable to breakdown using ultraviolet or sunlight and other chemical pathways [2]. In recent decades, there have been some reports on the metabolism of FPB by microbial degradation [10,11,12,13,14,15]; however, this degradation approach is not always practical in some cases, due to factors such as the inefficient efficiency of biodegradation, the greater impact of environmental conditions on the activity of the screened microorganisms, the high cost of using these microorganisms, and the long period required reach the degradation index of the herbicide residue. Therefore, it is necessary to develop innovative approaches to prevent environmental contamination of FPB from agricultural applications.

Photosensitizers refer to a class of molecules that transfer energy to the reactants via proton absorption in photochemical reactions, thereby promoting the degradation of the reactants. Common photosensitizers include inorganic compounds, metal ions, natural organic matters, surfactants, and some pigments. TiO_2_ is one of the most appropriate semiconductor materials to be employed as a photosensitizer. The photocatalytic properties of TiO_2_ are derived from the formation of photogenerated charge carriers (the so-called electron-hole pairs) which occurs upon the absorption of ultraviolet corresponding to the band gap [16]. Anatase-TiO_2_ is a wide band gap semiconductor with a band gap energy (Ebg) of 3.2 eV, which is equivalent to the energy of a photon with a wavelength of 387.8 nm [17,18]. When anatase TiO_2_ was irradiated by the light source with a wavelength of less than 387.8 nm, the electrons of TiO_2_ could be excited into the conduction band from the valence band to generate the electron-hole pairs. The hole has a greater reactivity and is the main component to carry the light quantum. The photogenerated holes in the valence band diffuse to the TiO_2_ particle surface and react with adsorbed water molecules to form hydroxyl radicals (•OH) with strong oxidability and can directly decompose organic pollutants. Meanwhile, electrons in the conduction band typically participate in reduction processes [19].

The nano-TiO_2_ was first employed to degrade organic pollutants in water [20]. Thereafter, photocatalytic oxidation by nano-TiO_2_ has attracted substantial attention as a water pollution control technology [21,22]. Currently, nano-TiO_2_ has become one of the most popular photocatalysts due to its effective photodegradation of the refractory organic compounds, its commercial availability due to being relatively inexpensive, its chemical stability under harsh conditions, the possibility of coating as a thin film on solid support, ease of preparation, and its highly oxidizing photogenerated holes. There is also abundant literature on the photocatalytic degradation of organic compounds [23,24,25,26,27,28,29,30,31].

**Figure 1:**
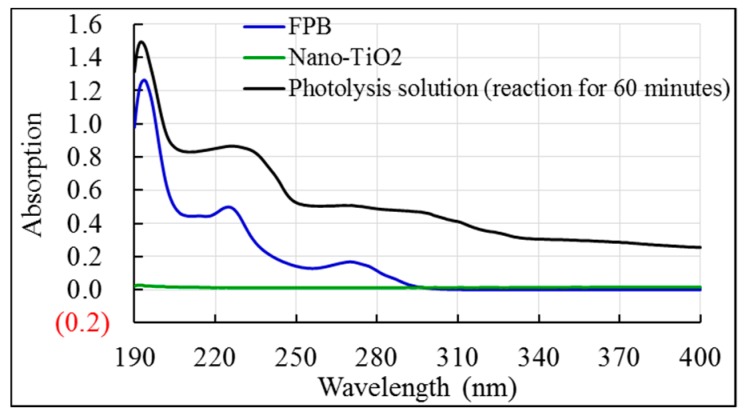
The ultraviolet absorption spectra of FPB (Fluazifop-p-butyl), nano-TiO_2_ and FPB photodegradation in the presence of nano-TiO_2_.

Photocatalytic degradation is an important non-biodegradation pathway of pesticides in the environment. The processes and products of photolysis have a great impact on the efficacy, metabolism, toxicity and environmental fate of pesticides. The action of shortwave radiation may chemically alter pesticides in the environment. The ultraviolet absorption spectrum of FPB shows the typical main maximum absorption at about 225 nm and 270 nm, respectively (Figure 1).

Derivatization of organic acids [32,33,34], phenol compounds [35,36] and alcohols [37,38,39], especially hydroxy acids and alcohols, to volatile derivatives is necessary prior to GC or GC-MS analysis. In addition, the solubility of these silicane derivatives in the low–polar solvents can be improved, thereby making them easier to perform GC or GC-MS analysis.

Nano-TiO_2_ was one of the most popular and efficient photocatalysts for photodegradation of organic pollutants. In order to rule out other interfering factors, an ingenious smear method was adopted to find a low-cost and environmentally friendly method to accelerate photodegradation of FPB in the environment.

## 2. Materials and Methods 

### 2.1. Chemicals and Equipment

The following chemicals and reagents were used in this study: FPB (94.86% purity, Dr. Ehrenstorfer, Augsburg, Germany); N, O-bis (trimethylsilyl) trifluoroacetamide (BSTFA), trimethylchlorosilane (TMCS) and nano-TiO2 powder (anatase, <25 nm particle size, and 99.9% purity) (Sigma-Aldrich, Duen, Germany); acetonitrile and n-hexane (HPLC grade) (Mreda Medical Technologies, Inc., Columbia, SC., USA).

### 2.2. Analytical Instruments

The photocatalytic degradation experiments were conducted under a rotary photochemical reactor (Nanjing xujiang electromechanical plant, China) equipped with a high-pressure mercury lamp (500 W, main emission line spectrum at 365 nm) and a xenotest instruments (Shanghai guangpin test equipment manufacturing Co., Ltd., China) equipped with a Xenon lamp (800 W/m2). The other instruments used in this study were as follows: Biotage TurboVAP^®^ LV Concentration Workstation (Zymark Corp., Boston, MA, USA), UV-2450 Spectrophotometer and UFLC system (Shimadzu Corporation, Kyoto, Japan), Agilent 7890A-5975C Inert XL GC-MS (Agilent Technology Inc., Santa Clara, CA, USA).

### 2.3. Experimental Procedure

#### 2.3.1. Optimization of Nano-TiO_2_ Dosage

The quartz slides (76 mm × 26 mm), which were smeared with 1 mL acetonitrile suspension of nano-TiO_2_ (containing 0.1 mg FPB, and 0, 2.5, 5, 10, 15, 20, 25 mg nano-TiO_2_, respectively), were placed under a xenon lamp and a high-pressure mercury lamp, respectively, for photocatalytic degradation experiments. The distance of the slides from the light source was 10 cm, and the temperature of the reaction system was maintained at 25 ± 1 °C. A control experiment for dark treatment was conducted by wrapping the slides in aluminum foil under the same conditions. After 60 min of illumination, the slides were ultrasonically eluted in 5 mL of acetonitrile for 2 min. Next, quantitative analysis of FPB in the eluent was performed on UFLC on a thermo BDS C_18_ column (4.6 mm × 250 mm, 5 µm) at a detection wavelength of 270 nm. FPB was eluted with acetonitrile/water (55:45, v/v) at a flow rate of 1 mL/minute. The column temperature was 30 °C. The injection volume was 10 μL. The limit of detection for FPB was 0.01 mg/L. The photodegradation rate was calculated with Equation (1):

(1) photolysis rate (%) = [(a−b)/a]×100

where *a* was the residual concentration of FPB in the dark control and *b* was the residual concentration of FPB in the light treatment.

#### 2.3.2. Photolytic Kinetics

The slides which were smeared with 1 mL acetonitrile suspension of nano-TiO_2_ (containing 0.1 mg FPB, and optimized dosage of nano-TiO_2_). After the organic solvent on the slides had evaporated, it received radiation of the xenon lamp and the high-pressure mercury lamp for 0, 20, 40, 60, 80, 100, 120, 150, 180, 240, 300 and 360 min, respectively. Afterwards, the samples were taken at different times to measure the residual concentration of FPB as the Section 2.3.1. Based on the obtained results, the first-order kinetic model (Equation (2)) was established and was used to depict the nano-TiO_2_ mediated photodegradation of FPB.

(2) $$\left[C_{t}\right]=[C_{0}]\;e^{-k\;t}$$

(3) $$T_{1/2}=\ln2/k$$

(4) $$Ef\;\left(\%\right)=\left[\left(k_{1}-k_{0}\right)/k_{0}\right]\times 100$$

where *t* was time, [*C_0_*] and [*C_t_*] were residual concentration at time zero and time *t* of light treatment, respectively. *k* was the photodegradation rate constant. *T*_1/2_ (half-life) of degradation was calculated from the rate constants (*k*). *Ef* was photosensitization efficiency, *k*_1_ was the reaction rate constant of FPB with nano-TiO_2_, and *k*_0_ was the reaction rate constant of FPB without nano-TiO_2_.

#### 2.3.3. Identification of Photolysis Products

In order to obtain enough photolytic products for trimethylsilane derivatization and the subsequent identification, the reaction system was scaled up based on the optimized dosage of nano-TiO_2_. After illumination of high-pressure mercury lamp for 60 min, the residues on the slides were ultrasonically eluted with 5 mL n-hexane. Next, 250 μL solution of the photolytic products was transferred into a 20 mL headspace vial (containing 250 μL of BSTFA + TMCS (99:1, v/v)) and allowed to undergo derivative reaction for 30 min at a constant temperature of 65 °C.

The trimethylsilane derivatives were identified by GC-MS on DB-642 fused silica capillary column (30 m × 0.25 mm, 1.4 µm). The oven temperature was started at 40 °C (held for 3 min), ramped at 5 °C/minute to 260 °C (held for 8 min). The temperatures of injector, quadrupole, ion source and the interface were set at 260, 150, 230 and 260 °C, respectively. Helium at a constant flow rate of 1 mL/minute and 50 mL/minute was used as carrier gas and make-up gas, respectively. Injections were made in split mode with a split ratio of 1:10. The MS was operated in electron impact mode with an ionization potential of 70 eV and the spectra were obtained at a scan range of m/z 50 to 500. The injection volume was 1 μL.

## 3. Results and Discussion

### 3.1. Optimized Nano-TiO_2_ Dosage

**Figure 2:**
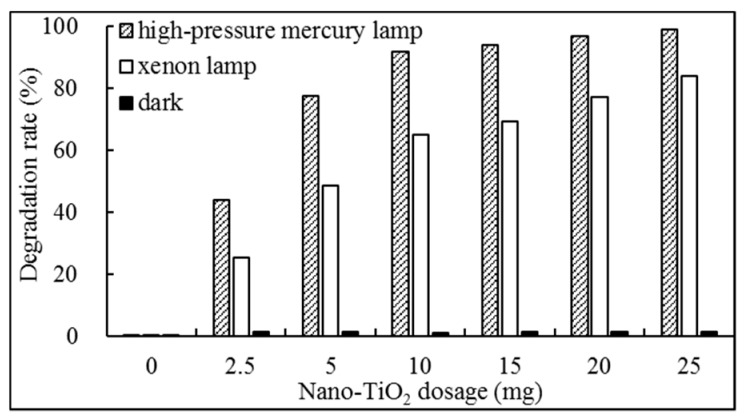
Effect of nano-TiO_2_ on photodegradation rate of FPB under the illumination of different light source.

Figure 2 showed that the photodegradation rate of FPB by the different dosage of nano-TiO_2_ mediated occurred in the following order: high-pressure mercury lamp > xenon lamp > dark. Furthermore, the maximum absorption wavelength of FPB is in the ultraviolet region (Figure 1). These facts indicated that the shortwave radiation of the high-pressure mercury lamp could easily facilitate the photodegradation of FPB. By contrast, the radiation of xenon lamp consists mainly of visible light with less shortwave. Consequently, the photodegradation of FPB may not occur as readily under radiation of a xenon lamp than under the emission of a high-pressure mercury lamp.

In addition, from Figure 2, it is not difficult to see that the photodegradation rate of FPB in 60 min depends on the dosage of applied nano-TiO_2_, and nano-TiO_2_ exhibited a clear photosensitizing effect on FPB photodegradation at lower dose range; however, the change of sensitization efficiency has begun to become insignificant when the dosage of nano-TiO_2_ increases from 10 to 25 mg in this photochemical reaction system. This may be due to the consequences of the light shielding by excess nano-TiO_2_. This indicated that the production of •OH by 10 mg nano-TiO_2_ was higher than less nano-TiO_2_. In general, nano-TiO_2_ was an effective photosensitizer in photodegradation of FPB. Therefore, the dose of nano-TiO_2_ was optimized at the value which was 1 mL acetonitrile suspension of nano-TiO_2_ (containing 0.1 mg FPB, 10 mg nano-TiO_2_) in the reaction system of nano-TiO_2_ mediated photodegradation of FPB.

### 3.2. Photolysis Kinetics and Photosesitization Efficiency

**Figure 3:**
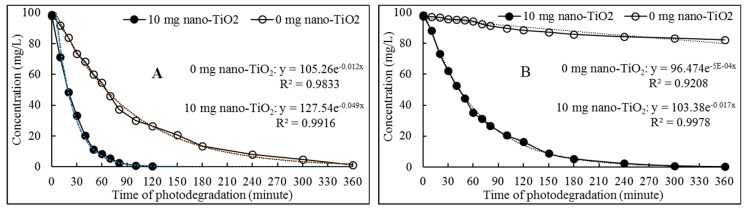
Kinetics of nano-TiO_2_ mediated photodegradation of FTB under high-pressure mercury lamp (**A**) and under xenon lamp (**B**) irradiation.

The residual concentration of FPB in the photocatalytic degradation system was accompanied by the first-order kinetics. (Figure 3). The photodegradation rate constant (0.049 min^−1^) of FPB with 10 mg nano-TiO_2_ under high-pressure mercury lamp irradiation was circa 4-fold than that (0.012 min^−1^) without nano-TiO_2_, and the constant (0.017 min^−1^) of FPB with 10 mg nano-TiO_2_ under xenon lamp irradiation was 34-fold than that (0.0005 min^−1^) without nano-TiO_2_. Based on the result, on the one hand we infer that the irradiation of high-pressure mercury lamp could be absorbed by FPB to accelerate its photodegradation, and on the other hand we have to recognize the strongly photosensitized FPB degradation by nano-TiO_2_ under both high-pressure mercury lamp and xenon lamp. However, the calculated *Ef* values (3300) by Equation (4) under xenon lamp irradiation was 8.8-fold than that (308) under high-pressure mercury lamp irradiation. Therefore, for nano-TiO_2_ mediated photosensitization degradation of FPB, the efficiency of xenon lamp irradiation is relatively higher than high-pressure mercury lamp.

From Equation (2), the calculated *T*_1/2_ of FPB by nano-TiO_2_ mediated under high-pressure mercury lamp and xenon lamp were 14.15 min and 40.77 min, respectively. However, in the absence of nano-TiO_2_, the calculated *T*_1/2_ of FPB under the two light sources was 57.76 min and 1386.29 min (theoretical calculation), respectively. These results also clearly indicated that nano-TiO_2_ exerts effective photosensitization on the photodegradation of FPB, whether it is under high-pressure mercury lamp or xenon lamp irradiation.

### 3.3. Identification of Photolysis Products

**Figure 4:**
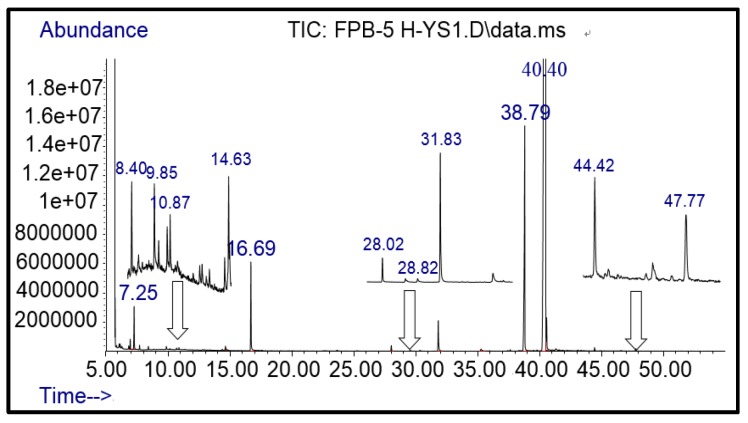
The Total Ion Chromatography (TIC) of derivatized photolytic products of FPB.

As mentioned above, the advanced oxidization process has been widely used in contaminant treatment due to being highly active and non-selective to different target compounds. However, it might encounter the recalcitrance from parent compounds. Thus, GC-MS measurements were carried out to elucidate the photocatalysis degradation products and degradation pathways of FPB by nano-TiO_2_ mediated. It was not difficult to see from Figure 4, with neat FPB and nano-TiO_2_ exposed to ultraviolet radiation for 1 h, and afforded at least twelve photolytic products. According to the MS analysis of the photolysis products of FPB in the total ion chromatogram (TIC), the twelve main photolytic products were identified and listed in Table 1.

Compared with the MS of FPB (compound 10, m/z 383), there was a fragment with m/z of 399 found, which might be ascribed to the remove n-butyl group (m/z 57) from FPB molecular and then the trimethylsilyl (m/z 73) connected with it by trimethylsilylation, and combining other characteristic ion fragments, the product was considered to be FP (compound 9, m/z 327). Subsequently, the fragment holding m/z of 283 was also detected, which was probably formed by the removing butyl acetate group (m/z 101) from FPB or removing carboxyl (m/z 57) from FP, it was identified as compound 5 (m/z 283). Furthermore, the fragment (m/z 310) probably derived from silicane derivatives of butyl 2-(4-hydroxyphenoxy)propanoate (m/z 238) which was formed by the fracture of the ether bond between pyridine ring and benzene ring in structure of FPB. The product was speculated to be compound 13 (m/z 238). In addition, the fragment with m/z of 471 may correspond to the result of hydroxylation of the pyridine ring or benzene ring of the FPB. It was inferred as compound 11 (m/z 399). In addition, the fragment with m/z of 385 probably derived from FPB, combining other characteristic ion fragments, the product was presumed to be 2-(4-(5-(trifluoromethyl) pyridine-2-yloxy)phenoxy)propan-1-ol (compound 12, m/z 313). The fragment holding m/z of 327 should be a trimethylsilyl derivative which formed from the trimethylsilylation of the structure which was losing the butyl propionate in FPB structure, and it was concluded to be FP (compound 8, m/z 255). Others, products with m/z of 254, 235, 147, 146, 130 and 93 were respectively assigned as hydroquinone (compound 7, m/z 110 = 254–73−73 + 1 + 1), 5-(trifluoromethyl)pyridin-2-ol (compound 3, m/z 163 = 235–73 + 1), 3-(trifluoromethyl)pyridine (compound 2, m/z 147), propionic acid (compound 6, m/z 74 = 146–73 + 1), butyl propionate (compound 1, m/z 130) and phenol (compound 4, m/z 93 = 165–73 + 1).

### 3.4. Photodegradation Pathway

**Figure 5:**
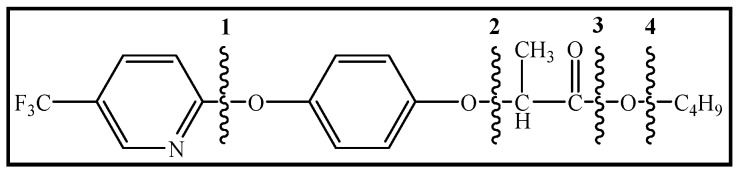
Possible cleavage sites for FPB molecules undergoing photocatalytic degradation.

**Figure 6:**
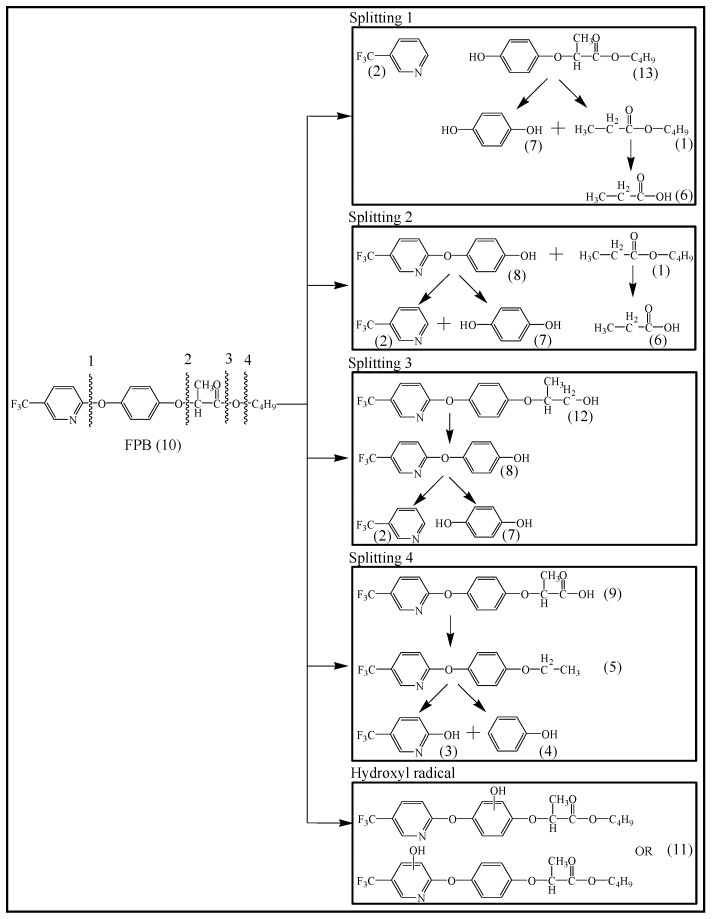
The proposed photocatalysis degradation pathways of FPB by nano-TiO_2_.

From the analysis of the molecular structure formula of FPB (Figure 5), it can be seen that the FPB molecular contains two ether bonds and one ester bond, all of which were relatively easy to break under high-pressure mercury lamp or xenon lamp irradiation. Combined with the speculated photolysis products (Table 1) of FPB by nano-TiO_2_ mediated, it was proposed that at least four degradation pathways and one reaction involving hydroxyl radicals exist in this degradation process, as shown in Figure 6.

## 4. Conclusions

In the photocatalytic degradation, the photosensitizers performed by transferring irradiation energy to some light-insensitive reactants to enhance their absorptive intensity or expand their spectra. The photosensitizing effect of nano-TiO_2_ was induced by irradiation sources with wavelength less than or equal to 387.8 nm. Therefore, nano-TiO_2_ can be used as an effective photosensitizer to accelerate the FPB photodegradation not only under high-pressure mercury lamps, but also when stimulated by the solar spectrum light source (xenon lamp light). From Figure 1, we can clearly see that the absorption of FPB degradation in the presence of nano-TiO_2_ becomes more intense and the absorption peak shifted toward long wavelength in the ultraviolet absorption spectra. Thus, the significantly broader and continuous spectrum of xenon lamp may be more conducive to light adsorption by nano-TiO_2_, which may explain why xenon lamp irradiation has a higher photosensitization efficiency than high-pressure mercury lamp. Furthermore, the xenon lamp in this study has the higher intensity, which also contributes to its enhanced photosensitization efficiency.

According to this study, it was considered that the ether bond and the ester bond of the FPB molecule were easily cleavable sites of the photocatalytic degradation. In addition, based on the photocatalytic reaction mechanism of nano-TiO_2_ combined with the identified photolytic products, it can be concluded that the nano-TiO_2_ mediated the photocatalytic degradation of FPB belongs to redox reaction involving hydroxyl radicals. Through a radical reaction, FPB in the environment can be effectively photosensitized degradation. The optimal dosage of nano-TiO_2_ was added in the photodegradation reaction system of FPB, and over time, it was expected to reduce the presence of FPB in the environment.

In the traditional photocatalytic reaction system, nano-TiO_2_ particles are too small to be particularly prone to loss, and the suspended particles in the solution compete with the nano-TiO_2_ to absorb the radiation energy, which affects the photocatalytic performance of the photocatalyst. In recent years, nano-TiO_2_ loading technology has increasingly entered the field of researchers. In this study, nano-TiO_2_ mediated the photocatalytic degradation of FPB was simulated by a coating method, and the results obtained indicate that this method is a beneficial attempt to the effective application of solid-phase nanocatalysis. This undoubtedly broadens the comprehension and recognition of nano-TiO_2_ as a photosensitizer.

In particular, considering the crucial importance of exogenous compounds for the healthy development of the ecological environment, the detailed ecotoxicological information of photolytic products of FPB is still an open question which must be considered in further studies.

## Figures and Tables

**Table 1:** Speculative photolytic products of FPB by nano-TiO_2_ mediated.

Compound No.	Retention Time (Minute)	Major Fragment Ion(m/z)	Speculative Structure and Precise Molecular Weight
1	7.25	57, 75, 130	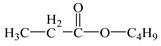 (130)
2	8.40	69, 78, 127, 147	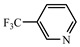 (147)
3	9.85	73, 96, 146, 163, 235	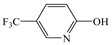 (163 = 235–73 + 1)
4	10.87	73, 66, 78, 94, 166	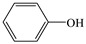 (93 = 166–73 + 1)
5	14.63	121, 146, 238, 254, 283	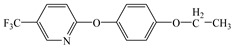 (283)
6	16.69	73, 74, 117, 146	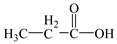 (74 = 146–73 + 1)
7	28.02	73, 81, 110, 165, 254	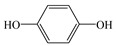 (110 = 254–73−73 + 2)
8	28.82	73, 146, 235, 238, 327	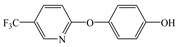 (255 = 327–73 + 1)
9	31.83	73, 146, 237, 282, 327, 399	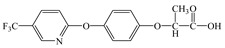 (327 = 399–73 + 1)
10	38.79	146, 238, 254, 282, 383	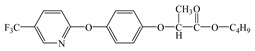 (383)
11	40.40	73, 145, 254, 282, 310, 383, 471	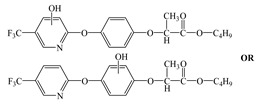 (399 = 471–73 + 1)
12	44.42	73, 131, 146, 238, 385	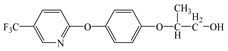 (313 = 385–73 + 1)
13	47.77	73, 129, 165, 181, 310	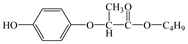 (238 = 310–73 + 1)
